# The hard steps model and whole Earth-system transitions

**DOI:** 10.1098/rstb.2024.0105

**Published:** 2025-08-07

**Authors:** Andrew Watson

**Affiliations:** ^1^ Global Systems Institute, University of Exeter, Exeter, UK

**Keywords:** biosignatures, astrobiology, evolution, exoplanets

## Abstract

The hard steps model is a ‘toy’ mathematical representation of evolution towards complex life on Earth or Earth-like planets. It assumes that, at the longest time scale, the rate of evolution towards increased complexity is governed by unlikely transitions that happen randomly and rarely. Applied to Earth, the model suggests a small number of such transitions in the pathway to ‘intelligent observers’—humans. The transitions are usually envisaged as occurring instantaneously, but this ignores the reality that on Earth, the evolution of life and the planetary environment have been inextricably linked. The critical steps should be seen as initiating whole Earth-system transitions that take hundreds of millions of years to complete, as in the events that caused, and followed after, the Paleoproterozoic and Neoproterozoic glacial episodes. These were both caused by, and drivers of, evolutionary advances that were necessary for complex life to arise. I adapt the model to include such delays and show that it then suggests just two or three hard steps to humans. The model predicts that the search for biosignatures in exoplanet atmospheres may find planets with Archean-like atmospheres, but probably will not find the signature of a planet with a Proterozoic or modern Earth-like atmosphere.

This article is part of the discussion meeting issue ‘Chance and purpose in the evolution of biospheres’.

## 1. Introduction

What kind of life, and how much of it, is present on other planets? Is microbial life common, perhaps ubiquitous throughout the Universe or at any rate in our galaxy, or is it rare? And what of more complex organisms and ‘intelligent’ observers such as ourselves? Are they out there, waiting for us to contact them, or are we alone? Versions of these questions have been asked ever since the Copernican revolution, when it became apparent that the Earth was not the centre of the Universe [1]. In the next few decades, we will likely start to get some answers to them, using observations of exoplanet atmospheres. Now seems a good time, therefore, to clarify hypotheses for what we might find, as these have the chance of being tested by such observations.

At present, the only planet that we know for sure has life on it is the Earth. In contrast to our complete ignorance about life elsewhere, we have accumulated a wealth of knowledge about the development of life on this planet, so it seems reasonable that we could begin to answer these questions by applying that knowledge. However, our existence as complex, big-brained animals is the result of a particular evolutionary pathway, so we cannot say whether a similar history would be followed on any broadly Earth-like planet that is the right distance from its star, or whether what has happened on Earth is very unusual, even unique (at least in the Milky Way Galaxy). One approach to dealing with this ‘observer self-selection’ bias [2,3] is given by the hard or critical steps model of evolution towards complexity, first introduced by Carter [4]. This is a ‘toy’ model, deliberately highly simplified from the real problem, in which the key assumption is that evolution towards complexity is paced by the occurrence of a few intrinsically unlikely steps that occur at random times. All other evolutionary events are assumed to occur sufficiently rapidly that they do not affect the long-term rate of increase of complexity. Clearly, this is a drastic simplification—there is no obvious rationale, for example, for dividing evolution into a few ‘hard’ steps while all the rest are ‘easy’. However, the model has some virtues that make it useful to study—it is mathematically tractable, its assumptions are few and easily stated, and it makes clear predictions that can be tested against observations. It has been used as a basis for a number of studies on the likely distribution and complexity of life in the Universe at large [5–9].

## 2. Basic properties of the model

**Figure 1.**
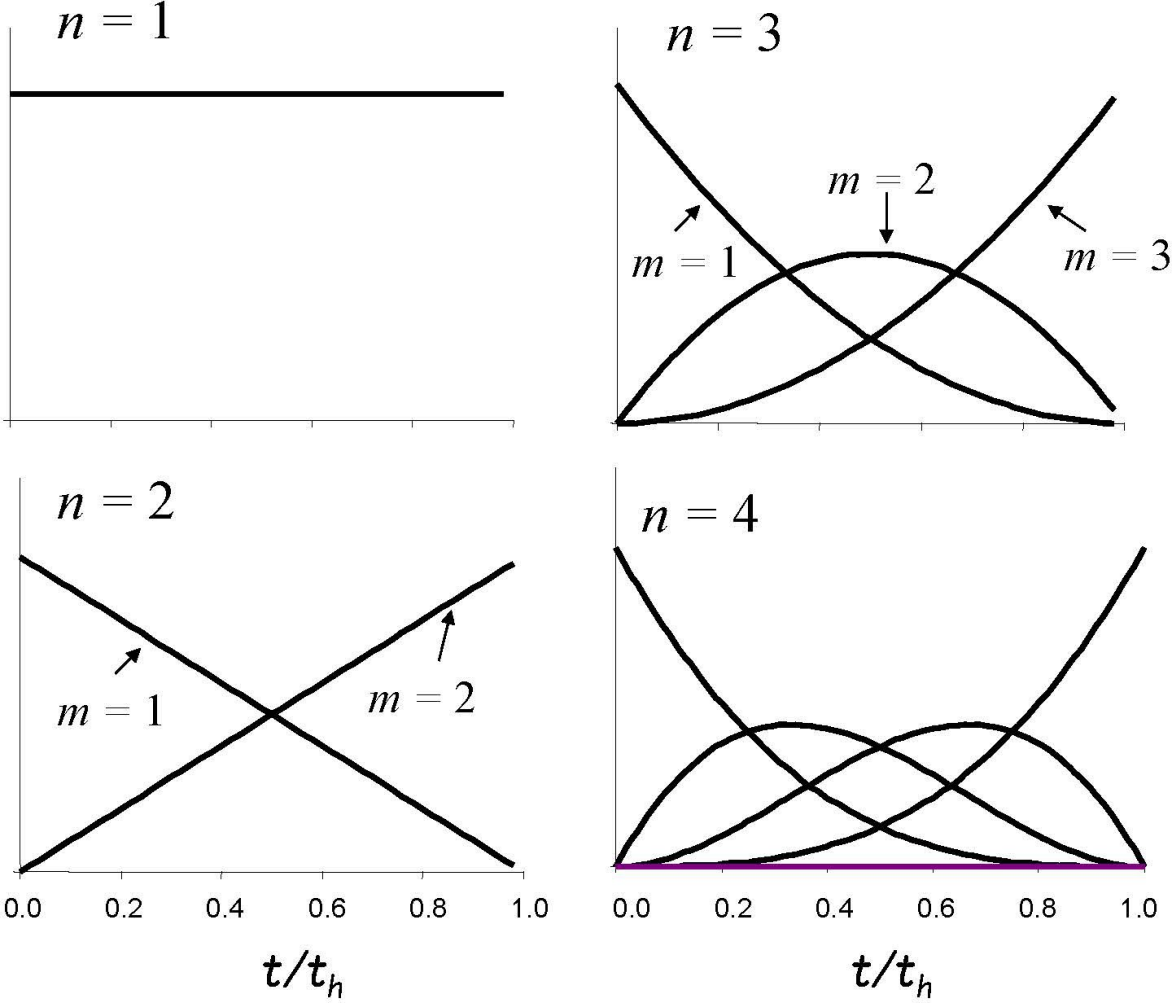
Probability distributions of the times of occurrence of the *m*th step in a sequence of *n* = 1, 2, 3 and 4 hard steps, plotted against time expressed as a fraction of the habitable lifetime, *t*_h_. The distributions are derived in Watson [9]. They have the form ${\left(t\right)}^{m-1}{\left(t_{\text{h}}-t\right)}^{n-m}$ . For sequences longer than one step, the last step is most likely to occur towards the end of the habitable lifetime.

To apply the model, we consider a population of planets on which conditions are suitable for complex life for a period *t*_h_*,* the ‘habitable lifetime’ of the planet. A sequence of *n* hard steps is assumed to be necessary in the path to evolution of the property of interest, which can in principle be any attribute of complex organisms. Each step can only occur after the previous steps in the sequence, after which it can occur with a uniform probability per unit time. This probability is very low, so that the expectation time for each step to occur, if there were no other constraints, would be longer than the habitable lifetime. On most planets, therefore, not even the first step is passed. However, if the population of planets is large enough, on a small subset, *n* steps will occur in the time available. Considering only that very small subset, we ask *when*, within the habitable lifetime, each step occurs. Probability distributions for the timing of each step in the subset of planets, where they all occur, have been derived [9] and examples are shown in figure 1 for values of *n* from 1 to 4. The steps tend to occur evenly spaced through the period *t*_h_ and for the *m*th step in a sequence of *n* steps, the expectation time is

(2.1) $$\left\langle t_{m}\right\rangle =\frac{mt_{h}}{\left(n+1\right)}.$$

However, as figure 1 shows, the probability distributions for the times at which the steps occur are quite broad, so in any individual case there could be substantial deviations from these times. For *n* > 1, the last step in a sequence tends to occur late in the habitable lifetime, and the larger *n* is, the later it is predicted to occur. These basic results hold regardless of the absolute probabilities of occurrence of individual steps, provided they are all small enough that they are unlikely to occur within a period *t*_h_.

While the model can be applied to any unlikely sequence of steps, the interesting application is to the evolution of humans on Earth—and by implication, to complex life and similarly intelligent organisms on other planets. According to the model, if we consider our emergence as the *n*th step in a sequence, the timing of our origin as a fraction of *t*_h_ can be used to make an estimate of the number of steps *n*. When Carter first proposed the model, he took *t*_h_ for Earth to be 10 billion years, this being the expected lifetime of the Sun on the main sequence. Since we have emerged 4.5 billion years after the formation of the Earth, this implied a best fit to equation (2.1) of *n* = 1, just one hard step in our evolution. However, subsequent studies of the long-term evolution of the Earth’s environment consistently suggest that the habitable lifetime of Earth is closer to 5 or 6 billion years than 10 billion years [10–14]. In that case, we have evolved more than three-quarters through the habitable lifetime, allowing for higher values of *n* between 3 and 6 for the case for humans on Earth.

## 3. Alternative models

The hard steps model explains the fact that complex life has evolved towards the end of the lifespan of the biosphere by postulating the existence of unlikely and difficult steps in that evolution. There could, of course, be other explanations for the late emergence of complexity on Earth that do not intrinsically assume it is unlikely. For example, evolution of complexity could require a large number of biological innovations, each of which takes time to occur, but each of which leads to the next in a very predictable progression. A related idea is that evolutionary innovations are highly convergent, with similar solutions to the challenges presented by the environment being repeatedly found and exploited by the process of evolution, so that similar types of organisms to those on Earth will be inevitable if life arises elsewhere [15]. Alternatively, it has been argued that there are no unlikely steps, but rather it is the long period needed to reshape the global environment to be suitable for complex life that is responsible for our relatively late appearance on Earth [16].

However, the hard steps model has several other features that make it attractive because they are consistent with our current knowledge:

(1) It leads naturally to the simplest explanation of the Fermi paradox—that we see no evidence for intelligent life elsewhere. Intelligence arises very rarely because only on a very few ‘lucky’ planets will technologically competent, complex organisms arise.
(2) It explains the order of magnitude co-incidence of *t*_h_, the habitable lifetime of the Earth and the time it has taken for an observer species (humans) to appear here, both of which are in the range 4−6 billion years. This follows even though the *unconstrained* expectation time for such a species—the length of time needed on average for it to evolve if conditions remained indefinitely favourable for life—is assumed to be much longer than the actual habitable lifetime of planets. On a ‘lucky’ planet such as Earth, when all the necessary steps do occur in the available time, they are usually completed near the end of its habitable period.
(3) It predicts that the most difficult stages in the path of evolution towards complexity and intelligence will be rather evenly distributed through the history of the Earth, which (as discussed further below) does fit what we know about Earth’s history.

## 4. Candidates for hard steps

Previous authors have considered a range of possible transitions in the history of life as candidates for hard steps along the path to human evolution. A popular starting point [7,9,17] has been the list of ‘major transitions’ in evolution due to Szathmary & Maynard Smith [18,19]. These authors originally proposed eight such transitions, and there has been much debate in the literature since that time about how major transitions should be defined [20]. However, there is no reason to suppose that they all would be critical steps, or conversely that all the critical steps are such transitions. Candidate events should be well spaced through Earth’s history. They should also have occurred only once on Earth, because, by definition, they are *a priori* very unlikely to occur—while one occurrence was necessary for us to be here, two occurrences would be unlikely in the extreme. These criteria mean that several of the major transitions discussed by Szathmary and Maynard Smith can be discounted as candidates. For example, several of them predate the origin of prokaryotes, so they must all have occurred very early in Earth’s history. Such a cluster of events would be inconsistent with the model if more than one of them was a hard step [9].

As a relevant aside, we can note that it is by no means certain that anything about the origin of life on Earth is particularly unlikely. It has been argued that the early establishment of life after the formation of the Earth suggests that biogenesis is commonplace [21]. Perhaps life begins relatively easily given the right environment. Alternatively, perhaps some version of Panspermia is correct: bacteria are able to survive interplanetary or interstellar journeys inside rocky debris and were seeded onto the early Earth. If either of these possibilities is correct, we might expect one day to find fossil evidence for life on Mars, and perhaps evidence for still-extant life deep under the ice of Europa.

**Figure 2.**
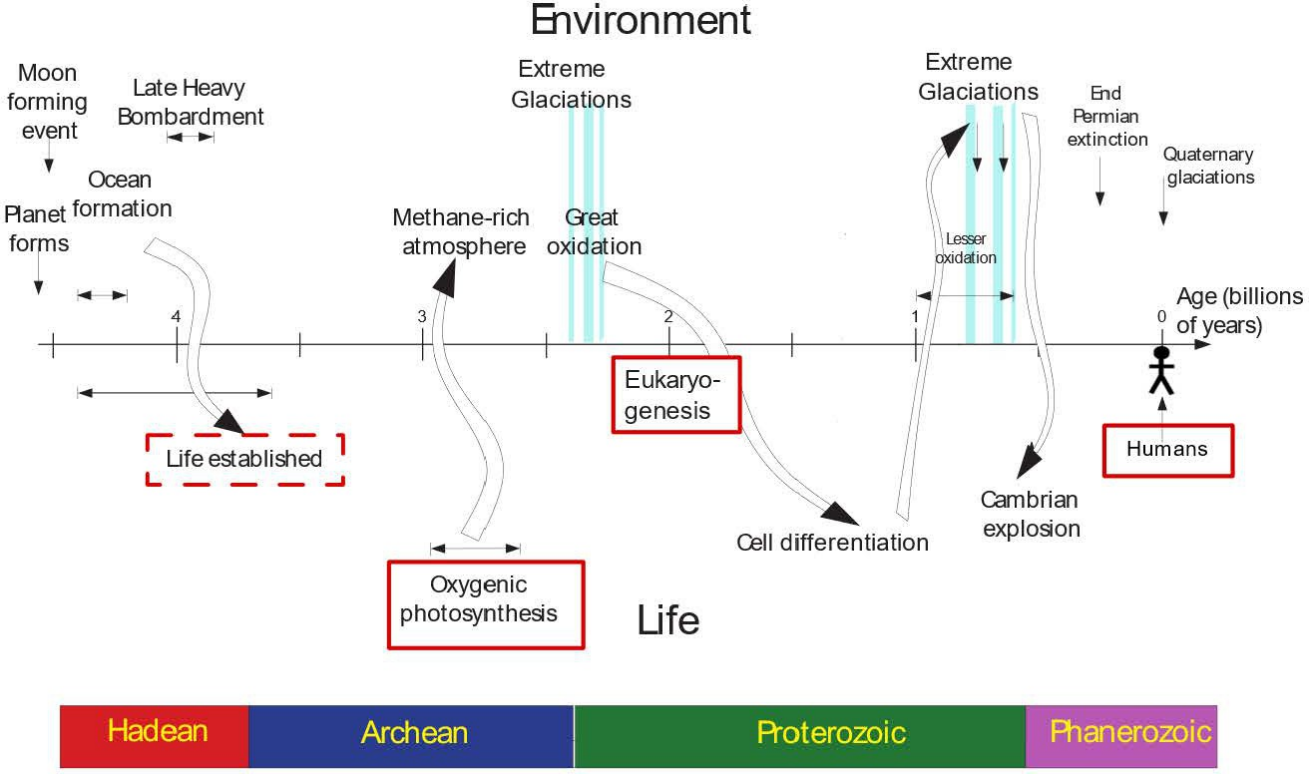
Cartoon showing some of the connections between evolutionary innovations (below the timeline) and changes in the Earth’s environment (above the line). Possible candidates for hard steps are outlined in red; the dashed box around ‘Life established’ reflects the uncertainty discussed in the text about whether or not biogenesis is an unlikely event. The figure is redrawn from Lenton & Bloh [12].

Suggestions for candidate hard steps in the literature have so far sought them only in the biological evolutionary history of life. However, in the past century, it has become increasingly clear that the evolution of life on Earth has not taken place on an unchanging planet, as if on a passive stage waiting for life to perform its drama. The fundamental insight of Lovelock’s Gaia theory, and before him of Vernadsky [22,23], is that Earth’s history is a co-production between the environment of the Earth’s surface and the life developing on it. The major transitions on Earth are not solely biological events, but combined biological, geological and climatological re-organizations. These transitions mark major phases in Earth’s history where the surface environment was transformed, with results that we today recognize in the major boundaries of geological time—the Archean, Proterozoic and Phanerozoic Eons (figure 2). In the context of the hard steps model, this insight leads us to ask whether the events surrounding those transitions could be candidates for hard steps? Are they unlikely transformations, or a likely sequence, liable to be repeated on many other planets? While the model as formulated to date treats hard steps as instantaneous point events, the transitions in Earth’s history are more protracted. They take time to occur, and the simple model does not account for this. Below, I consider what is known about the causes of these transitions and whether the transitions themselves, and their ultimate causes, fit the criteria for hard steps. I then adapt the mathematical model to include delays and show that this reduces the number of steps that it predicts to have occurred in deep time. The long-term consequences of the remaining steps can plausibly be identified with those Earth-system transitions.

### (a) The Archean–Proterozoic, the Great Oxidation Event

About 2.45 billion years ago (Ga), in the ‘Great Oxidation Event’ (GOE), oxygen, which had previously been only a trace component of the atmosphere at or below the part per million level, rose quickly in concentration by several orders of magnitude, probably to approximately 1% of the atmosphere [24]. At about the same time, the Earth experienced several periods of extreme glaciation. In some of these, ice extended close to the Equator [25] and the glacial strata are overlain by cap carbonates, indicating potentially ‘snowball’ events [26]. There has been some difficulty in determining whether the oxidation events preceded or followed the glaciations, but recent work suggests that initial oxidation occurred first [27]. It is believed that the oxidation and the glaciations are causally linked, with greenhouse gases such as methane and other hydrocarbons being suppressed to low concentrations by photolytically catalysed reaction with oxygen. Oxidation of the atmosphere was ultimately due to the invention by ancestral cyanobacteria of oxygenic photosynthesis, an innovation that, however. occurred well before the GOE, there being evidence for transient oxygen events in the atmosphere up to 2.9 Ga [28]. The delayed oxygenation of the atmosphere is predicted to have occurred because the Archean Earth was a reducing environment where oxygen had a short lifetime in the atmosphere. For O_2_ from photosynthesis to build up in the atmosphere, oxidation of the surface environment was first necessary, and until this occurred, the net effect of enhanced biological productivity may have been to increase methane and other reducing gas concentrations [29,30] rather than oxygen. The resulting enhancement of hydrogen escape due to increased methane production [31], and a declining net flux of reduced matter from the Earth’s interior to the surface, eventually tipped the balance to allow oxygen to increase, and once it passed a threshold of a few parts per million in the atmosphere, this increase would have been rapid and become permanent [32]. Oxygenic photosynthesis was essential for the formation of an oxidizing atmosphere and the delay between its evolution and the GOE can be considered to be the length of time required for it to take effect, given the particular circumstances at that time on Earth.

Because of this delay, the process of oxidation of the atmosphere does not fit the description in the naive hard steps model. However, the invention of oxygenic photosynthesis itself is a good candidate for a hard step. It occurred only once and is absolutely necessary for complex organisms to exist on the modern Earth. Though anoxygenic photosynthesis exists in a number of bacterial clades, the oxygenic variety, which requires the coupling of photosystems I and II with, in addition, a unique catalyst to enable the splitting of water molecules, is only possessed by the cyanobacteria among prokaryotes. All eukaryote organisms that are capable of photosynthesis (land plants and several different classes of algae) acquired that ability through endosymbiosis with chloroplasts that derive either primarily or secondarily from the bacterial source. A defensible hypothesis, therefore, is that oxygenic photosynthesis was an unlikely innovation. If so, even on planets that host bacterial life, most will not undergo an event analogous to the Great Oxidation that ended the Archean and ushered in the Proterozoic Eon.

### (b) Proterozoic–Phanerozoic transition

In the late Proterozoic, the Earth was again subject to a series of severe glaciations, for two of which (the Sturtian, at about 715 Ma, and the Marinoan at about 635 Ma) we have evidence for ice sheets formed close to the equator, so these may have been ‘snowballs’, possibly with a narrow belt of open water near the equator [33]. The causes of the glaciations are not agreed upon, but disturbances in the carbon cycle were extreme at this time [34,35] and low concentrations of CO_2_ in the atmosphere are thought to have played an important role. Suggested causes fall broadly into biological and geophysical camps: the case for biology is that the proliferation of life, including diversification of eukaryotes [36], and especially the evolution of complex multicellular fungi and lichen associations [37], could have allowed colonization of continents leading to increased rock weathering and drawdown of CO_2_ [38,39] or a buildup of organic carbon sequestered from the atmosphere [40]. Suggested geophysical causes include accelerated weathering of CO_2_ due to the breakup of the supercontinent Rodinia [25,41,42] or a concentration of continental areas in the tropics [43].

If biological causes are accepted as the main driver of this cooling of the planet, can we identify a root cause? We would have to point to the origin of eukaryotes as the key event, which was initiated much earlier, sometime shortly after the Paleoproterozoic glaciations and the Great Oxidation. Eukaryogenesis is often considered to be the most important transition towards complexity in the history of life [44]. An oxygen-rich environment was an essential precondition: the vast majority of eukaryotes require oxygen and possess mitochondria, originally acquired by endosymbiosis of bacteria, and needed for their oxygen metabolism. The first fossil evidence for eukaryotes appears shortly after the GOE, and acritarch body fossils begin to be found at approximately 1.7 Ga. The elaboration of eukaryotes then seems to have been a slow process stretching over a great length of time, with simple multicellular forms appearing in the mid-Proterozoic and the complex multicellular clades of animals, fungi and algae diverging at around 800 Ma [45,46]. Complex multicellularity has sometimes been cited as a hard step, but since it occurred independently several times within the eukaryotes [46] it does not itself fit the criterion of being exceptionally unlikely.

The origin of eukaryotes might qualify as a hard step. However, they arise comparatively soon after the GOE [47], so we can ask if eukaryote evolution was really so unlikely: might it not turn out to be a near-inevitable consequence of the environmental conditions and pre-existing evolutionary developments? To answer this question, it would help if we had an agreed model of eukaryote origins, but we do not; it remains a controversial area [48]. There is increasing evidence for a substantial number of endosymbiosis events contributing to eukaryogenesis, but this might be explained if the critical innovation was the evolution of phagocytosis [49], the ability of cells to feed by engulfing prey, which is unique to eukaryotes.

Immediately following the final glaciation of the Neoproterozoic (the Gaskiers, which was not global), the Ediacaran period marks the first appearance of large metazoans. The enigmatic Ediacarans lasted for about 60 million years before the rapid appearance in the fossil record of many different animal phyla at the start of the Cambrian, the dawn of the modern Phanerozoic world. Though complex multicellularity evolved independently several times, a case can be made that the animal multicellularity that developed so spectacularly at this time is special: the number of different cell types in animals is orders of magnitude greater than for plants or algae, and Cavalier-Smith argues that animal multicellularity was particularly difficult to evolve [50]. Metazoan architecture requires a unique degree of co-dependence of the constituent cells, with the great majority of the tens or hundreds of cell types relinquishing the ability to reproduce or obtain nutrients.

The association of the rise of animals with the Neoproterozoic extreme glaciations is suggestive, and it has been proposed that the extreme climate could have promoted the evolution of these ‘altruistic’ traits by restricting life to small refugia, genetically isolated from one another. This would allow small populations with variable founder members to develop in novel and ultimately productive directions without invasion by non-altruistic ‘cheats’ disrupting the early stages [51]. Severe climatic upheavals, providing they are not so serious that they kill all the complex organisms, may therefore be important in promoting increased complexity. Extreme climate upheavals also provide a reset for the biosphere, promoting mass extinction that leaves open niches for new types of organisms to colonize, a mechanism that is clearly apparent in the aftermath of more recent and better documented mass extinction events of the Phanerozoic, where the fossil record is more informative about the consequences.

In summary, an argument can be made that the origin of eukaryotes was a hard step, which was followed by a delay of greater than 1 Gyr during which eukaryotes diversified, eventually giving rise to algae, fungi and proto-animals. The algae and fungi formed lichen-like associations that colonized and weathered the continents, drawing down atmospheric CO_2_. This led to global-scale glaciations that stimulated further rapid diversification of life and so, eventually, to the modern world inhabited by large and complex animals.

## 5. Including Earth-system delays in the hard steps model

The discussion above leads to the appreciation that there may have been very substantial delays between key biological events and the massive changes that they eventually caused in the Earth system. We can identify a delay of order 0.5 Gyr between the evolution of oxygenic photosynthesis and the GOE, and of order or greater than 1 Gyr between the evolution of eukaryotes and the changes towards the end of the Proterozoic that their diversification enabled. To simulate this, the basic hard steps model can be modified to include ‘forbidden’ periods after some or all of the steps, during which the next step is not able to occur. A simple mathematical treatment of this is outlined in the electronic supplementary material to this article, where I show that when such periods are included, the expectation time for the last step in a sequence of *n* steps constrained to occur in a habitable period of *t*_h_ is given by

(5.1) $$\left\langle t_{n}\right\rangle =\;\frac{nt_{\text{h}}+\Delta }{\left(n+1\right)}.$$

Here, *Δ* is the cumulative total of the forbidden periods associated with the *n −* 1 previous steps. This expression can be compared to equation (2.1) with *n = m,* which is the equivalent for the original model—it is identical except for the addition of *Δ* in the numerator.

Applied to the case of humans on Earth, if the cumulative delay in previous steps is approximately 1.5 Gyr, this significantly changes the implications of the model. For example, if we take the emergence of humans to have occurred at 0.8*t*_h_, (implying *t*_h_ is about 5.7 Ga), the best fit to the original model is that there are *n* = 4 steps to humans. By contrast, in the revised model with *Δ* = 1.5 Gyr, the best fit is between 2 and 3. Since one of these events is, by definition, the emergence of humans in the geologically recent past, this suggests that there were most likely just one or two hard steps in deeper time. A self-consistent scenario is that there were two such steps, oxygenic photosynthesis and eukaryogenesis, which led to Earth-system changes that cumulatively took more than a billion years to reshape the planetary environment to the degree necessary for the further steps to occur.

## 6. Summary: pros, cons and predictions from the hard steps model

I conclude that the hard steps model remains a useful and viable framework for understanding the evolution of complexity in the Earth’s biosphere. Its prediction that there are a few unlikely, widely spaced events in the history leading up to humans is consistent with our understanding of Earth’s history, but it is obvious that the actual history of the Earth-life system is much richer than the simplistic model allows. In the model, the only brake on the rate of development of complexity is the need for the rare, randomly timed hard steps, whereas in reality, there are lengthy time delays due to slower geological, climatic and evolutionary changes that eventually transform the planet. We can identify the origin of oxygenic photosynthesis and most probably also the origin of eukaryotes as good candidate hard steps, each of which led eventually to wholesale changes of the entire Earth system, including the climate and composition of the oceans and atmosphere. There were in each case delays of hundreds of millions of years from the inception until these changes were sufficiently complete that the next step could occur. In the pathway to humans, we can also count as an unlikely step the origin of the combination of characteristics that separate us from our close animal relatives, since there seems no fundamental reason why a technologically able species could not have arisen at any time in the last 500 million years. After adapting the model to account for Earth-system delays, we suggest that the best fit for the case of humans on Earth is that there are just two hard steps further back in Earth’s history.

However, alternatives to the model can also be defended. In particular, we could explain the slow evolution of complexity by appealing only to the slow but predictable pace of environmental, geological and biological processes, rather than unlikely evolutionary happenstance [16]. These two perspectives make very different predictions about what will be found in the search for biosignatures on exoplanets. From the hard steps model, we would predict the following:

(1) If all life is rare, we will find no biosignatures. However, as noted above, there is no compelling evidence from Earth’s history that biogenesis is rare—in fact, the opposite has been argued, in which case prokaryote biospheres might be relatively common, and may even be found elsewhere in the Solar System—on Europa for example.
(2) However, oxygenic photosynthesis is a good candidate hard step, so we would predict that Proterozoic or Phanerozoic-like biospheres will be rare and we will almost certainly not find them.

The alternative, in which there are no hard steps, predicts that most planets on which life begins, if they have a sufficiently long habitable lifetime, will evolve oxidizing atmospheres and productive biospheres powered by photosynthesis. We might argue, therefore, that the detection of atmospheric oxygen or ozone in an exoplanet atmosphere would falsify the hard steps hypothesis. Caution is necessary, however, because some abiotic reactions also produce significant oxygen [52], as might abundant hydrogen escape [53,54]. It will be important, therefore, to search for evidence also of trace reducing gases such as methane, that would indicate a high degree of thermodynamic disequilibrium in the atmosphere—also a consequence of active photosynthesis. This combination was documented in the Earth’s infrared spectrum by the Galileo spacecraft [55] and, as pointed out by Hitchcock and Lovelock in the paper that first suggested life detection by atmospheric analysis [56], it would be a strong indication of a thriving biosphere.

## Data Availability

Supplementary material is available online [57].
